# Simulation and Experiment of Tilted Fiber Bragg Grating Humidity Sensor Coated with PVA/GO Nanofiber Films by Electrospinning

**DOI:** 10.3390/s25237386

**Published:** 2025-12-04

**Authors:** Li Deng, Hao Sun, Jiawei Xi, Yanxin Yang, Xin Liu, Chaochao Jian, Xiang Li, Jinze Li

**Affiliations:** 1 School of Optoelectronic Engineering, Xidian University, Xi’an 710071, China; 2 School of Intelligent Medicine, Shaanxi University of Chinese Medicine, Xi’an 712046, China

**Keywords:** tilted fiber Bragg grating, optical fiber sensor, graphene oxide, electrospinning

## Abstract

Relative humidity (RH) and temperature are crucial parameters in environmental monitoring and have attracted significant attention. However, traditional commercial sensors typically suffer from inherent limitations such as structural complexity, bulkiness, and high manufacturing costs. To address these issues, this study proposes a novel tilted fiber Bragg grating (TFBG)-based optical fiber humidity sensor, coated with a composite film of polyvinyl alcohol (PVA) and graphene oxide (GO). First, the sensing mechanisms of the TFBG functionalized with nanofiber films were theoretically analyzed, and the transmission spectra of TFBG under varied structural parameters were simulated. These theoretical investigations laid a solid foundation for subsequent experimental validation. Subsequently, PVA/GO composite nanofiber films tailored for humidity sensing were fabricated by electrospinning technology, and the proposed TFBG sensor was experimentally implemented in accordance with the theoretical design. The experimental results indicate that the developed sensor exhibits a humidity sensitivity of −0.24 pm/%RH within the RH range of 35–85%. Furthermore, we calculated temperature and RH changes while discounting cross-sensitivity, thereby enabling simultaneous decoupling of temperature and RH measurements. Owing to its distinctive advantages of compact size, light weight, and cost-effectiveness, the proposed TFBG sensor holds great promise for practical applications in environmental monitoring.

## 1. Introduction

Optical fiber sensors offer significant advantages, including small size, light weight, and immunity to electromagnetic interference [1], which have driven high market demand and promising development prospects. Various types of optical fiber sensors have been developed, such as TFBG sensors [2,3,4,5], distributed optical fiber sensors [6,7,8], and surface plasmon resonance (SPR) biochemical sensors [9,10,11,12]. Recent cutting-edge applications [13], represented by smart photonic wristbands for pulse wave monitoring, further demonstrate their versatility in wearable sensing scenarios. Specifically, TFBG is fabricated by inscribing gratings tilted along the fiber axis at a specific angle, resulting in a periodic refractive index (RI) distribution within the fiber core. This configuration allows light in the optical fiber core to couple to the cladding, enabling the detection of environmental parameter changes.

RH is a crucial parameter in environmental monitoring, with applications spanning food processing, storage rooms, agriculture, and even human comfort and health [14,15,16,17,18,19,20,21,22,23]. With the advancement of science and technology, the need for accurate and rapid RH detection has grown increasingly critical in both industrial production and daily life. To achieve optical fiber humidity sensing, a hygroscopic material is coated onto the optical fiber surface. This material expands and alters its refractive index upon absorbing moisture. By analyzing the transmission spectra of the optical fiber sensor, the environmental RH can be effectively detected.

Polyimide (PI) [24], Poly(methyl methacrylate) (PMMA) [25], Chitosan ((C_6_H_11_NO_4_)_n_) [26], and PVA/Poly(ethylene glycol) (PEG)/agar/Hydromed D4 [27] polymer coating layer have been utilized in optical fiber humidity sensors. As shown in Table 1, PI is coated on the FBG sensor as a moisture-sensitive material, which undergoes reversible volume expansion. But it requires higher temperatures for electrospinning. PMMA is coated on the POF surface for distributed humidity sensing, offering strong measurement capability and good stability. But the response time of this sensor is slower than most other humidity sensors. Chitosan has abundant amino and hydroxyl groups in its porous structure. But it is difficult to dissolve in general organic solvents and requires chemical treatment to obtain nanofibers. Dias Bernardo et al. compared the moisture-sensitive properties of four polymer materials on FPI and LPFG. PEG can employ a thick coating. But it was not possible to retrieve any information from the spectra of the PEG FPIs below the deliquescence relative humidity (DRH). Hydromed D4 exhibits optical stability, and the sensing response is low. Agar shows higher RI changes in the DRH area but requires heating to 80 °C to become a gel. Overall, PVA shows properties suitable for RH sensing.

Most hygroscopic polymers exhibit good coating properties (adhesion, uniformity, and thickness) and can be used to form thin films; however, they are limited by the structure of the material itself, which limits their hygroscopic ability. PVA nanofibers are one-dimensional nanomaterials known for their high specific surface area, high porosity, and tunable functionality [28]. The formula of PVA is (C_2_H_4_O)_n_, which is rich in -OH groups and is easy to spin. At high relative humidity, the blend has higher water sensitivity. Graphene is rich in native oxygen-containing groups, such as hydroxyl, carboxyl, epoxy, and carbonyl. To enhance RH sensitivity, we combined PVA polymer with 2D nanomaterial GO, which is rich in oxygen-containing groups, exhibits dispersibility and hygroscopicity, and can insert polymers between the GO layers. The water molecules absorbed by this PVA/GO composite film fill the void between PVA and GO and expand. Therefore, this study uses a mixed film of PVA and GO to enhance moisture sensitivity and improve sensor sensitivity.

Nanofibers can be fabricated through various methods, including electrospinning, templating, drawing, and self-assembly [29]. The principle of electrospinning involves spraying a solution under a strong electric field. The droplet at the needle tip transforms from a sphere into a cone (known as a “Taylor cone”) and extends into nanofibers [30]. This method can produce polymer nanofibers with a nanometer diameter range [31]. In 2019, Jiang et al. [32] demonstrated a GO-deposited tilted fiber grating structure that presented humidity response sensitivities of 18.5 pm/%RH and 0.027 dB/%RH. In 2020, Egor I. Dolzhenko et al. [33] presented a new technique of depositing PVA coating with an airbrush on the cylindrical surface of an optical fiber with real-time spectral monitoring, which was used for TFBG-assisted fiber hygrometer fabrication. In 2021, Wang et al. [34] proposed a TFBG sensor coated with a GO/multi-walled carbon nanotube hybrid nanomaterial. In 2022, Cheng et al. [35] explored a new method for developing temperature and relative humidity optical fiber sensors based on SPR, which were coated with carboxymethyl cellulose and polydimethylsiloxane for the simultaneous measurement of RH and temperature. In 2023, Feng et al. [36] proposed an excessively tilted fiber grating (Ex-TFG)-based humidity sensor with SnO_2_ and PVA hybrid materials. In 2024, Ren et al. [37] proposed a simple, reversible, and highly sensitive sensor with excellent linearity and stability in response to temperature and relative humidity, for the first time, by electrospinning a layer of polyvinyl alcohol nanofiber films coated on a microtapered long-period fiber grating (MT-LPFG).

Nanofibers have been widely employed as sensing elements for RH measurement, given their excellent sensing performances. However, a systematic analysis integrating both simulation and experimental results for tilted fiber Bragg grating (TFBG)-based sensors remains underexplored. In this work, we propose a novel TFBG-based RH sensor coated with electrospinning PVA/GO nanofiber films, where PVA/GO fibers are included in the PVA/GO composite films (of micro thickness) obtained on the surface of the optical fibers. We performed comprehensive simulation analyses and experimental response tests to systematically evaluate sensing performance.

## 2. Sensor Simulation

### 2.1. Operating Principle

**Figure 1:**
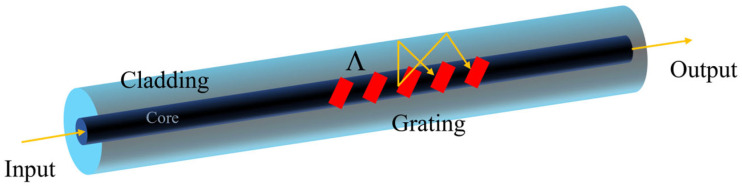
A schematic diagram of the TFBG sensor.

Figure 1 shows the structure of the TFBG sensor. The grating planes are angled by a certain degree to the fiber axis.

When the grating tilt angle is small, the TFBG is a reflective grating. There are two main coupling modes in the TFBG. One is the coupling between the forward transmission and backward transmission core modes, which exhibits a Bragg resonance peak at the long wavelength of the transmission spectrum. The other is the coupling between the forward transmission core modes and the backward transmission cladding modes, which exhibits a series of cladding mode resonance peaks at the short wavelength of the transmission spectrum. These two coupling modes are controlled by phase-matching conditions:

(1) $$\begin{array}{c} \beta_{\mathrm{core}}+\beta_{\mathrm{core}}=\frac{2\pi }{\Lambda }\cos\theta \\ \beta_{\mathrm{core}}+\beta_{\mathrm{clad}}=\frac{2\pi }{\Lambda }\cos\theta \end{array}$$

where $\beta_{\mathrm{core}}$ is the core mode propagation constants, and $\beta_{\mathrm{clad}}$ are the cladding mode propagation constants.

The resonant wavelengths $\lambda_{\mathrm{Bragg}}$ and $\lambda_{\mathrm{clad},i}$ of the core and cladding modes in the TFBG are determined by the phase-matching condition:

(2) $$\begin{array}{c} \lambda_{\mathrm{Bragg}}=\frac{2n_{\mathrm{eff},\mathrm{core}}\Lambda }{\cos\theta }\\ \lambda_{\mathrm{clad},i}=\frac{\left(n_{\mathrm{eff},\mathrm{clad},i}+n_{\mathrm{eff},\mathrm{core}}\right)\Lambda }{\cos\theta } \end{array}$$

where $n_{\mathrm{eff},\mathrm{core}}$ is the effective refractive index of the core mode, and $n_{\mathrm{eff},\mathrm{clad},i}$ is the effective refractive index of the *i*th order cladding mode.

The resonant wavelength of the core and cladding modes is related to the grating period and the effective refractive index of the core and cladding modes.

### 2.2. TFBG Parameter Simulation

OptiGrating (ver. 4.2.3.74) software was utilized to simulate the TFBG. The simulation model consists of four layers: the core, cladding, film, and external environment form. Initially, the main parameters of the grating, such as tilt angles, periods, and lengths, were simulated to determine the influence of their impact on the TFBG transmission spectrum. Subsequently, the TFBG sensor coated with nanofiber films was simulated to identify the optimal parameters for experimental testing. The simulation parameters are consistent with those of the experiments.

From the theoretical analysis in Section 2.1, it can be seen that the TFBG tilt angle, period, and length influence the core mode and the cladding modes. The transmission spectra of TFBG humidity sensors coated with nanofiber films with different tilt angles were simulated.

**Figure 2:**
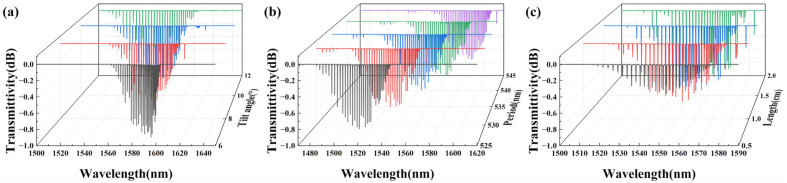
TFBG simulation spectra of different (**a**) tilt angles; (**b**) periods; (**c**) lengths.

The refractive index of the core and cladding were set to 1.4682 and 1.4628, respectively, with radii of 4.1 μm and 62.5 μm. The refractive index of the environment and nanofiber films were set to 1 and 1.4847, with thicknesses of 20 μm and 0.5 μm. The grating modulation depth was set to 0.001 (Δn*_mod_*/n*_eff_*). The following describes simulations of the TFBG with different tilt angles, periods, and lengths, respectively:

(1) The TFBG tilt angle was set to 6°, 8°, 10°, and 12°. The TFBG length and period were set to 1 cm and 535 nm. The simulated transmission spectra of different tilt angles are shown in Figure 2a. It can be seen that as the TFBG tilt angle increases, the core mode and cladding modes shift toward the long-wavelength direction. The distance between the core mode and the cladding modes increases, and more cladding modes appear, broadening the transmission spectrum. The tilt angle of the 8° spectrum exhibits a stronger core mode and more cladding modes, so we selected the TFBG tilt angle of 8°.
(2) The TFBG period was set to 525 nm, 530 nm, 535 nm, 540 nm, and 545 nm, with a tilt angle of 8° and a length of 1 cm. The simulated transmission spectra of different periods are shown in Figure 2b. As the grating period increases, both the core mode and cladding modes shift toward the long-wavelength direction, while the transmission depth remains similar. This characteristic allows for the adjustment of grating periods to position the transmission spectrum within a specific band. We selected the TFBG period of 535 nm to make the central wavelength of the transmission spectrum 1550 nm.
(3) The TFBG length was set to 0.5 cm, 1 cm, 1.5 cm, and 2 cm. The TFBG tilt angle and period were set to 8° and 535 nm. The simulated transmission spectra of different lengths are shown in Figure 2c. As the grating length increases, the transmission depth increases. However, an excessively long grating length can compromise the mechanical strength of the optical fiber. To balance mechanical strength and transmission depth, we selected a grating length of 1 cm, ensuring both high mechanical strength and a deep transmission spectrum.

### 2.3. TFBG Sensor Simulation

Suppose we have two materials: Material A has a refractive index of $n_{a}$, and material B has a refractive index of $n_{b}$. These two materials can be combined to produce a composite material. The refractive index of this composite material is simply the weighted average α of the refractive index of the two base materials, as shown in the following formula:

(3) $$index=n_{a}\alpha +n_{b}(1-\alpha )$$

The refractive indices of PVA and GO are 1.463 and 2.388, and the α is 0.919. The calculated refractive index of the dry PVA/GO composite film is 1.538. However, the refractive index of the composite film varies with different humidity. The corresponding relationship between the film refractive index and RH is shown in Table 2.

#### 2.3.1. TFBG Humidity Simulation

**Figure 3:**
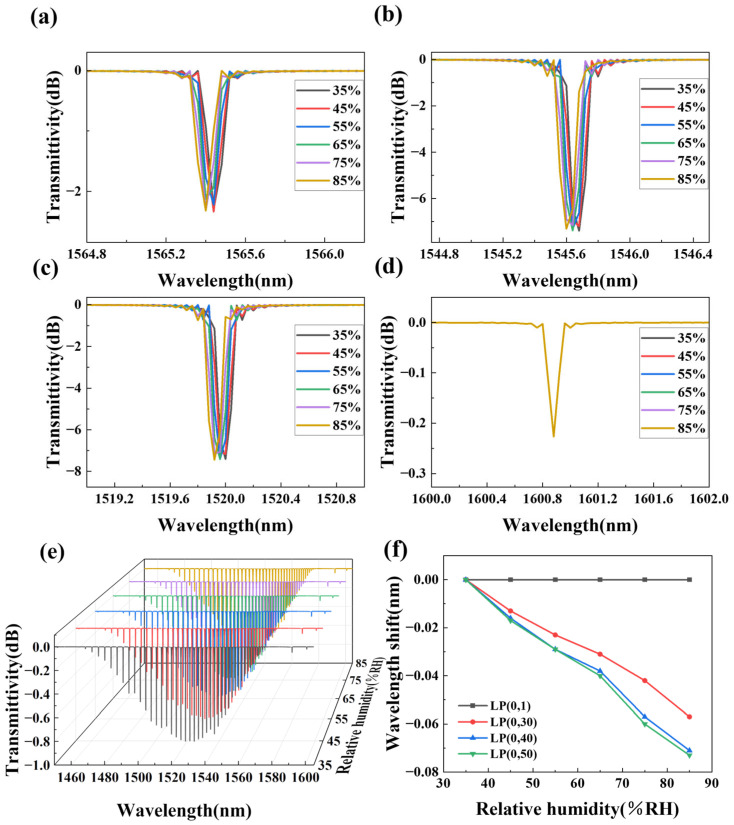
(**a**) The cladding mode LP (0, 30); (**b**) the cladding mode LP (0, 40); (**c**) the cladding mode LP (0, 50); (**d**) the core mode LP (0, 1); (**e**) TFBG simulation spectra in different RH; (**f**) the wavelength shift in different RH.

Next, the TFBG sensor coated with nanofiber films was simulated under different humidity, as shown in Figure 3e. The TFBG tilt angle, length, and period were set to 8°, 1 cm, and 535 nm, and other parameters were set as above. The refractive index of the composite film is provided in Table 2, and the film thickness was set to 0.5 μm.

The TFBG has weak guiding characteristics, and the transmission of light has linearly polarized (LP) modes. The longest cutoff wavelength mode is the lowest mode, called the fundamental mode LP (0, 1), and all other modes are high-order modes LP (0, n). To research the sensing characteristics of the different orders, three cladding modes of LP (0, 30), LP (0, 40), LP (0, 50), and core mode LP (0, 1) in different wavelengths were selected for analysis. The spectra are shown in Figure 3a–d. As humidity increases, the cladding modes of each order shift toward the short-wavelength direction, and the core mode position does not change.

The spectra shift of each mode at different RH levels is shown in Figure 3f. The core mode remains constant at 0, indicating its insensitivity to changes in the external environment refractive index. The spectral shifts of the cladding modes LP (0, 30), LP (0, 40), and LP (0, 50) are different. The spectral shift of the high-order cladding mode LP (0, 50) is larger, indicating that the higher the order of the cladding mode, the more sensitive it is to the refractive index changes in the external environment. The wavelength sensitivity of the LP (0, 50) is −1.44 pm/%RH in the range of 35–85%RH.

#### 2.3.2. TFBG Temperature Simulation

**Figure 4:**
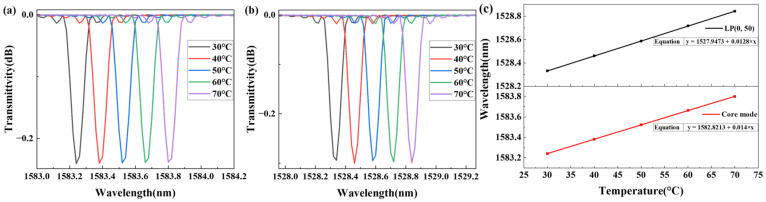
(**a**) Core mode spectra at different temperatures; (**b**) cladding mode LP (0, 50) spectra at different temperatures; (**c**) fitting curves of the wavelength and temperature.

The core mode and cladding mode LP (0, 50) spectra at different temperatures, recorded every 10 °C in the range of 30–70 °C, are shown in Figure 4a,b. The thermal expansion coefficient and thermo-optical coefficient of the core and cladding were set to 5.5 × 10^−7^/°C and 8.3 × 10^−6^/°C, respectively. The wavelength of the core and cladding mode shifts towards the longer-wavelength direction as the temperature increases. The thermal expansion coefficient and thermal–optical coefficients of the TFBG change with temperature, causing the wavelength shifts, which make the sensor sensitive to temperature.

The temperature data are fitted in Figure 4c. The LP (0, 50) spectral shift sensitivity is 12.8 pm/°C, and the core mode spectral shift sensitivity is 14 pm/°C. The spectral shifts of the core and cladding modes are nearly identical.

## 3. Materials and Methods

### 3.1. Electrospinning

The preparation steps of the PVA/GO composite solution were as follows: Firstly, 0.7 g of GO was added to 100 g of deionized water and subjected to ultrasonic dispersion for 0.5 h. Secondly, the GO solution was mixed with 8 g of PVA powder and magnetically stirred at a speed of 600 r/min for 2 h until a homogeneous solution was formed. Finally, a PVA/GO composite electrospinning solution with a PVA mass fraction of 8.7% was obtained.

**Figure 5:**
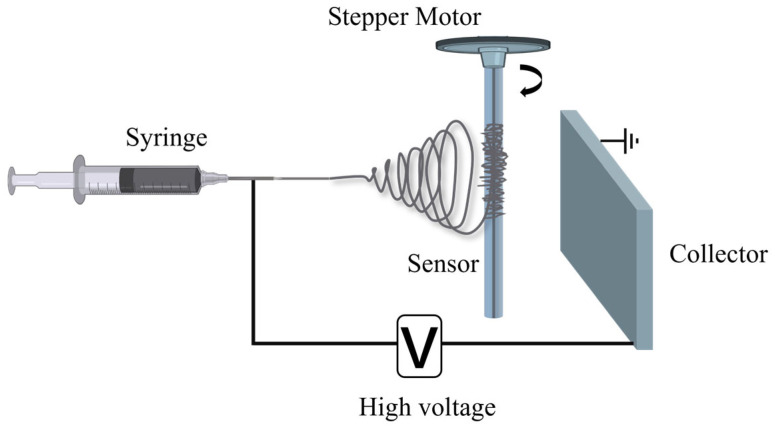
Schematic diagram of PVA/GO nanofibers electrospinning.

The schematic diagram of PVA/GO composite nanofibers wrapped around the TFBG by electrospinning is shown in Figure 5. The electrospinning apparatus was purchased from YUNFAN-DP30 (Tianjin, China). The optical fiber sensor was fixed to a stepper motor to maintain stability and verticality. Subsequently, the PVA/GO composite solution was transferred into a syringe equipped with a blunt needle (19G), which was then mounted on a propulsion platform. A voltage of 18 kV was applied to the needle, and the syringe pump was set to a feeding rate of 0.0015 mm/s for 2 min. Through this process, PVA/GO composite nanofibers were wrapped around the optical fiber sensor.

### 3.2. Characterization of TFBG Sensor

**Figure 6:**
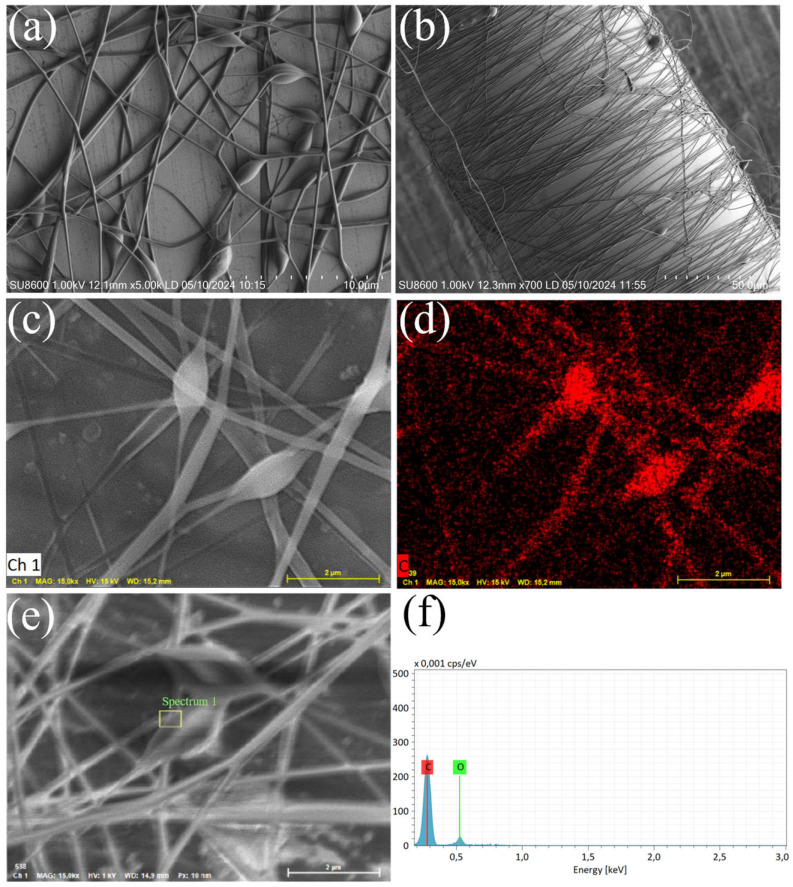
(**a**,**b**) SEM of PVA/GO nanofibers on TFBG; (**c**,**d**) energy spectrum scanning mapping; (**e**,**f**) EDS of PVA/GO nanofibers.

Figure 6a,b present SEM images of the porous network morphology of PVA/GO nanofibers, which are tightly and uniformly wrapped around the optical fiber surface. The nanofibers have an average diameter of ~200 nm, and the film thickness is approximately 0.5 μm. Figure 6c,d show energy-dispersive spectroscopy (EDS) mapping of the PVA/GO nanofibers. Among these, Figure 6d displays the distribution of carbon (C) in red; the ellipsoidal particles exhibit high C content, indicating that graphene oxide (GO) nanoparticles are encapsulated within the PVA nanofibers. Figure 6e,f show EDS spectra of the PVA/GO nanofibers. The results show that C accounts for ~80% and oxygen (O) for ~20% of the total elements, further confirming the presence of GO nanoparticles in the nanofibers.

## 4. Experiment Results and Discussions

**Figure 7:**
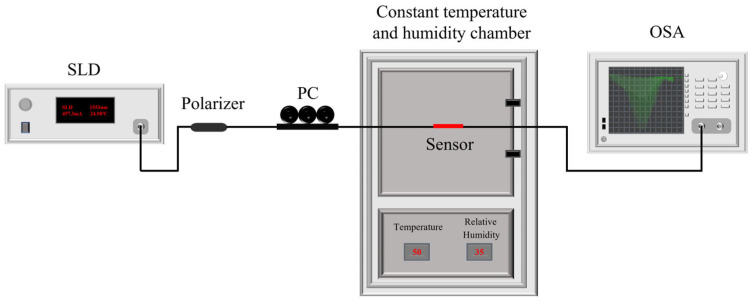
Experimental test system.

The experimental setup shown in Figure 7 includes the TFBG (tilted angle 8°, period 535 nm, and length 1 cm), PVA (AR, Xilong Scientific, Shantou, China), GO (AR, Suiheng Technology, Shenzhen, China), broadband source (SLD-1550, Thorlabs, Shanghai, China), optical spectrum analyzer (OSA) (AQ6370B, Yokogawa, Tokyo, Japan), and constant temperature and humidity chamber (BPS-50CL, Bluepard Instruments, Shanghai, China). Between the broadband source and the optical fiber sensor, there is a polarizer and a polarization controller (PC, Thorlabs, Shanghai, China), which enable the control and adjustment of the polarization of light traveling within the TFBG with either TE (s-polarized) or TM (p-polarized) signals.

### 4.1. Humidity Test

**Figure 8:**
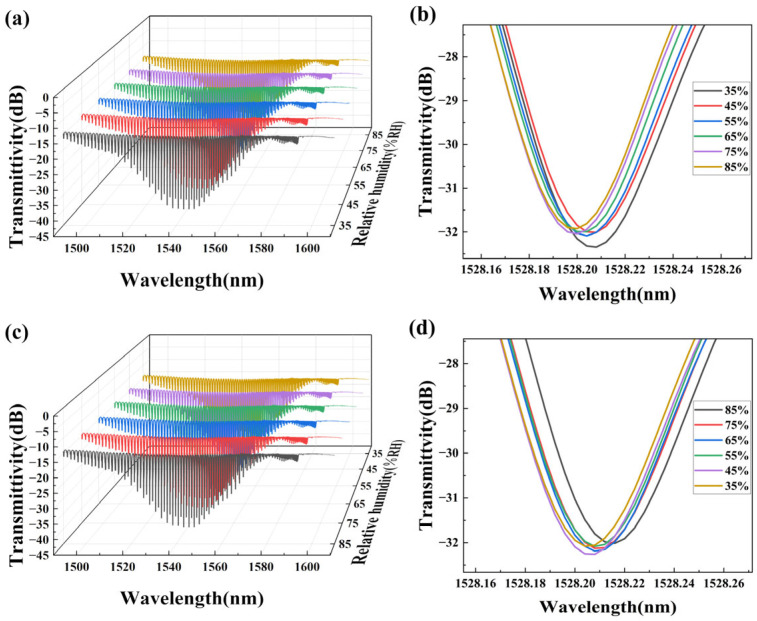
TFBG transmission spectra: (**a**,**b**) RH increases; (**c**,**d**) RH decreases.

The temperature was set to 50 °C, and the humidity was increased from 35%RH to 85%RH. The spectra of the TFBG sensor were recorded every 10%RH by OSA. Based on the simulation results, the LP (0, 50) cladding mode was selected for analysis. Figure 8a–d show the spectral variation of the TFBG coated with PVA/GO nanofibers when humidity changes. As shown in Figure 8b, it can be found that as the humidity increased, the cladding mode spectra shifted toward the short-wavelength direction. Subsequently, the humidity was decreased from 85%RH to 35%RH. As shown in Figure 8d, as the humidity decreased, the LP (0, 50) cladding mode spectrum shifted toward the long-wavelength direction.

**Figure 9:**
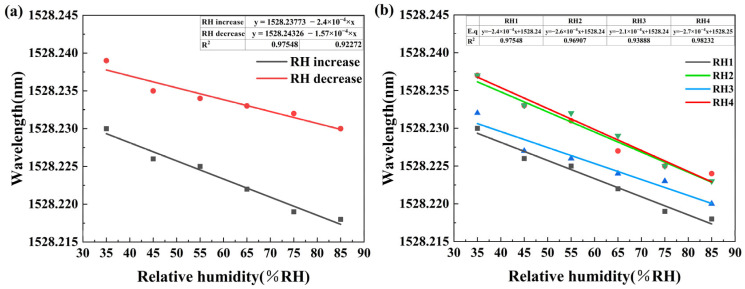
(**a**) Fitting curves of wavelength and RH; (**b**) repeatability test of sensor humidity response.

The fitting curves for increasing and decreasing RH are shown in Figure 9a. It can be calculated that the RH increased sensitivity was −0.24 pm/%RH, and the RH decreased sensitivity was −0.16 pm/%RH. The differences in the fitting curves were primarily due to the humidity hysteresis characteristics of the PVA/GO nanofiber films. The humidity-sensitive materials of humidity sensors have different reaction speeds and degrees during water molecule adsorption and desorption, resulting in inconsistent responses during RH increases and decreases.

### 4.2. Repeatability Test

Repeatability is an important indicator for evaluating sensor performance. To demonstrate the sensor repeatability and stability, we conducted a humidity response test every two days with three repetitions. The results are shown in Figure 9b. RH1 corresponds to the first sensor fabricated, while RH2, RH3, and RH4 are additional sensors manufactured using the same fabrication method for repeatability tests. The sensitivities of the three tests were −0.26 pm/%RH, −0.21 pm/%RH, and −0.27 pm/%RH, respectively. The experimental results show that after four repeated tests, the sensor continued to function normally with good repeatability. The measurement results of the three tests were close.

### 4.3. Temperature Test

**Figure 10:**
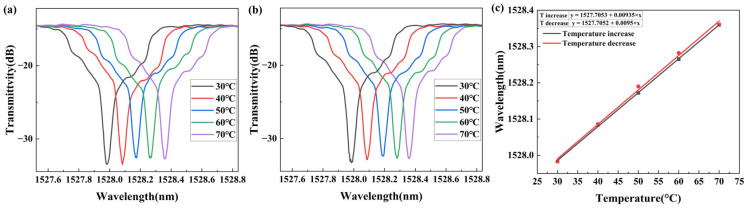
TFBG spectral response of samples coated with PVA/GO nanofibers under different temperatures: (**a**) as temperature increases; (**b**) as temperature decreases; (**c**) wavelength–temperature fitting curves.

Temperature response tests were carried out as shown in Figure 10. The LP (0, 50) cladding mode was also selected for analysis in the temperature range of 30–70 °C. As the temperature increased, the spectra shifted toward the long-wavelength direction. Conversely, as the temperature decreased, the spectra shifted toward the short-wavelength direction. The fitting curves of increasing and decreasing temperature are shown in Figure 10c. The average spectral shift sensitivity was 9.425 pm/°C.

### 4.4. Temperature Compensation

**Figure 11:**
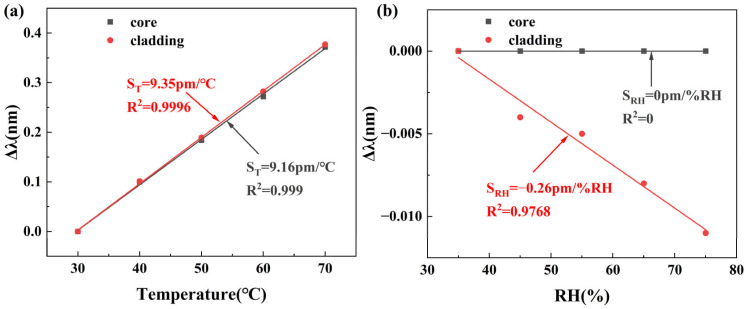
Wavelength shifts of core mode LP (0, 1) and cladding mode LP (0, 50). (**a**) Temperature; (**b**) RH.

We carried out temperature and RH measurements to record TFBG transmission spectra. The temperature varied from 30 °C to 70 °C in steps of 10 °C, with a constant RH of 50%. The humidity varied from 35%RH to 85%RH in steps of 10%RH, with the temperature constant at 50 °C. The wavelength shifts are shown in Figure 11. The core mode LP (0, 1) is located at 1596 nm, and the cladding mode LP (0, 50) is at 1528 nm.

Core mode LP (0, 1) and cladding mode LP (0, 50) demonstrate a sensitivity of 9.16 pm/°C and 9.35 pm/°C to temperatures, respectively. For RH, cladding mode LP (0, 50) is sensitive to RH changes, and the fitting curves show the sensitivity of −0.26 pm/%RH, while the core mode LP (0, 1) spectral shift is always 0, indicating that the core mode is not sensitive to RH changes.

The matrixial method of sensitivities is used as a way to distinguish between the contributions of different parameters in the sensor’s spectrum by measuring the changes in two different spectral regions, with different responses to both parameters [26]. In this research, the parameters are temperature and RH. Temperature and RH are denoted as ΔT and ΔRH. Since both the temperature and RH response are linear, their wavelength shifts are $\Delta \lambda_{core}$ and $\Delta \lambda_{cladding}$, separately, which can be given by: $\Delta \lambda_{core}=S_{T,core}\Delta T+S_{RH,core}\Delta RH$ and $\Delta \lambda_{cladding}=S_{T,cladding}\Delta T+S_{RH,cladding}\Delta RH$, where $S_{T,core}$ and $S_{RH,core}$ are core mode LP (0, 1) sensitivities to temperature and RH, and $S_{T,cladding}$ and $S_{RH,cladding}$ are cladding mode LP (0, 50) sensitivities to temperature and RH. The sensitivity measured results are shown in Figure 11. $S_{T,core}$ and $S_{RH,core}$ are 9.16 pm/°C and 0 pm/°C, and $S_{T,cladding}$ and $S_{RH,cladding}$ are 9.35 pm/°C and −0.26 pm/%RH. According to the matrixial method of sensitivities, substituting the measurement results, we obtain the following matrix:

(4) $$\left[\begin{array}{c} \Delta T\\ \Delta RH \end{array}\right]=\frac{1}{-2.431}\left[\begin{array}{cc} -0.26 & 0\\ -9.16 & 9.35 \end{array}\right]\left[\begin{array}{c} \Delta \lambda_{FBG}\\ \Delta \lambda_{\min} \end{array}\right]$$

where −2.431 is the calculated determinant. Using Equation (4), we can calculate temperature and RH changes while eliminating cross-sensitivity, thereby enabling simultaneous decoupling of temperature and RH measurements.

Table 3 lists the performance indicators of optical fiber grating humidity sensors. The comparison shows that the sensor proposed in this research can provide satisfactory sensitivity and range, and more importantly, the sensor has been tested in simulation and experimental environments, with good stability and repeatability.

In summary, OptiGrating software was used to simulate the transmission spectra of TFBG under different humidity and temperature conditions. The higher-order cladding mode was more sensitive to the refractive index change of the external environment. Therefore, the LP (0, 50) mode was selected for humidity and temperature measurements. In the experiment, we combined the electrospinning material of PVA with GO to fabricate PVA/GO composite nanofiber films. The ultra-large specific surface area of nanofibers was utilized to improve the performance of the sensor. While optical fiber dispersion, environmental interferences, and other factors were not considered in the simulation analysis, leading to differences between the simulated and measured spectra, the experimental data still largely validate the simulation results. In the future, we will explore the following three targeted strategies to enhance the sensor’s RH sensitivity and fabricate high-performance humidity sensors: (1) optimization of the PVA/GO composite, (2) regulation of PVA/GO nanofibers, and (3) sensitivity enhancement through SPR or lossy mode resonance (LMR).

## 5. Conclusions

In this paper, we propose a tilted grating humidity sensor coated with electrospinning PVA/GO nanofiber films, supported by simulation and experimental results. In the simulation, the TFBG transmission spectra under different grating parameters and environmental temperatures and humidity were simulated by OptiGrating software. In the experiment, we combined PVA with GO to improve the performance of the sensor. The humidity sensitivity of the sensor is −0.24 pm/%RH in the range of 35–85%RH. The proposed humidity sensor offers advantages, such as simple manufacturing, low cost, and stability, presenting a novel method for environmental monitoring, which can be effectively applied to real-time environmental monitoring in precision manufacturing environments, non-invasive humidity detection in food packaging, and high-sensitivity humidity sensing in biomedical devices.

We used the matrixial method of sensitivities to calculate temperature and RH changes. However, the matrixial method of sensitivities is valid only within a restricted temperature range around 50 °C. In future work, we will further conduct experiments to characterize the RH sensitivity over a broader temperature range. This will enable us to confirm whether S_RH_ remains stable or requires temperature-dependent adjustments, thereby validating the matrix correction method for broader practical applications.

## Figures and Tables

**Table 1:** Other optical fiber humidity sensors based on polymer coating layers.

Sensor	Coating Material	Advantage	Disadvantage	Refs.
FBG	Polyimide	Reversible expansion	Higher T required	[24]
POF	PMMA	Stability	Slow response time	[25]
TFBG	Chitosan	Amino/carboxyl-rich	Hardly soluble	[26]
FPI/LPFG	PVA/PEG/agar/Hydromed D4	PEG: thick coating. Hydromed D4: optical stability. Agar: higher RI changes. PVA: suitable for RH sensing.	PEG: No information below DRH. Hydromed D4: low sensing response. Agar: needs heating.	[27]

**Table 2:** Refractive index of composite film in different RH [38,39].

RH	35%	45%	55%	65%	75%	85%
**RI**	1.4847	1.4832	1.4812	1.4792	1.4772	1.4749

**Table 3:** Comparison with other RH sensors.

Sensor Structures	RH Sensitivity	Detecting Range	Coating Material	Refs.
FBG	0.02 nm/%RH	15–95%	Di-ureasil	[40]
FBG	0.01 nm/%RH	20–80%	GO	[41]
FBG	2.4 pm/%RH	38–62%	OR	[42]
TFBG	0.045 dB/%RH	45–75%	PVA	[43]
TFBG	0.00317 nm/%RH	20–70%	PAHP4	[44]

## Data Availability

The raw data supporting the conclusions of this article will be made available by the authors on request.
